# GBM heterogeneity as a function of variable epidermal growth factor receptor variant III activity

**DOI:** 10.18632/oncotarget.12600

**Published:** 2016-10-12

**Authors:** Olle R. Lindberg, Andrew McKinney, Jane R. Engler, Gayane Koshkakaryan, Henry Gong, Aaron E. Robinson, Andrew J. Ewald, Emmanuelle Huillard, C. David James, Annette M. Molinaro, Joseph T. Shieh, Joanna J. Phillips

**Affiliations:** ^1^ Department of Neurological Surgery, Brain Tumor Center, University of California, San Francisco, CA, USA; ^2^ Touro University California, College of Osteopathic Medicine. Vallejo, CA, USA; ^3^ Departments of Cell Biology, Oncology, and Biomedical Engineering, School of Medicine, Johns Hopkins University, Baltimore, MD, USA; ^4^ Université Pierre et Marie Curie (UPMC) UMR-S975, Inserm U1127, CNRS UMR7225, Institut du Cerveau et de la Moelle Epiniere, Paris, France; ^5^ Department of Neurological Surgery, Feinberg School of Medicine, Northwestern University, Chicago, IL, USA; ^6^ Helen Diller Family Comprehensive Cancer Center, University of California, San Francisco, CA, USA; ^7^ Epidemiology and Biostatistics, University of California, San Francisco, CA, USA; ^8^ Institute for Human Genetics, Department of Pediatrics, University of California, San Francisco, CA, USA; ^9^ Department of Pathology, Division of Neuropathology, University of California, San Francisco, CA, USA

**Keywords:** RTK activity, extracellular matrix, tumor heterogeneity, vessel co-option, invasion

## Abstract

Abnormal activation of the epidermal growth factor receptor (EGFR) due to a deletion of exons 2-7 of EGFR (EGFRvIII) is a common alteration in glioblastoma (GBM). While this alteration can drive gliomagenesis, tumors harboring EGFRvIII are heterogeneous. To investigate the role for EGFRvIII activation in tumor phenotype we used a neural progenitor cell-based murine model of GBM driven by EGFR signaling and generated tumor progenitor cells with high and low EGFRvIII activation, pEGFR^Hi^ and pEGFR^Lo^. *In vivo*, *ex vivo*, and *in vitro* studies suggested a direct association between EGFRvIII activity and increased tumor cell proliferation, decreased tumor cell adhesion to the extracellular matrix, and altered progenitor cell phenotype. Time-lapse confocal imaging of tumor cells in brain slice cultures demonstrated blood vessel co-option by tumor cells and highlighted differences in invasive pattern. Inhibition of EGFR signaling in pEGFR^Hi^ promoted cell differentiation and increased cell-matrix adhesion. Conversely, increased EGFRvIII activation in pEGFR^Lo^ reduced cell-matrix adhesion. Our study using a murine model for GBM driven by a single genetic driver, suggests differences in EGFR activation contribute to tumor heterogeneity and aggressiveness.

## INTRODUCTION

Glioblastoma (GBM) is the most common primary malignant brain tumor in adults and is characterized by intertumoral and intratumoral heterogeneity [1–4], including differences in genomic and epigenomic alterations [5, 6], invasive phenotype [7], and RTK signaling pathway activity [8]. Although gene expression analysis of bulk tumor tissue suggests tumors can be divided into discrete transcriptionally-defined subgroups [6, 9], the analysis of multiple samples from the same tumor frequently reveals striking intratumoral diversity in gene copy number, mutations, and stem cell signature [2–4, 10]. GBM heterogeneity endows this cancer with an ability to adapt to and evade the anti-tumor effects of nearly any therapy. Thus, despite the development and application of targeted therapeutics based on an improved understanding of GBM molecular characteristics [11–15], tumor heterogeneity continues to confound efforts for improving GBM treatment outcomes.

The most common RTK alteration in GBM is of the epidermal growth factor receptor (EGFR) and altered EGFR signaling is considered a driver of malignant characteristics in up to 45% of tumors. EGFR is frequently amplified in GBM [11] and approximately 20-30% of amplified tumors express a constitutively active variant of EGFR, EGFRvIII or EGFR*, which contains a deletion of exons 2-7 [11, 16–20]. Several cancers are driven by altered EGFR signaling, yet associations between total receptor levels, activation levels, and clinical outcome have been mixed [21–23]. In GBM, increased expression of EGFR or EGFRvIII are also not clearly associated with decreased overall survival [18, 24–27] and a link between EGFR activation and outcome in GBM is yet to be determined.

EGFR signaling is important to the normal biology of neural stem cells (NSCs), and promotes the transition from a quiescent to an activated state [28]. In the murine postnatal brain, EGFR expression in NSCs is reduced upon neuronal lineage commitment. Aberrant activation of EGFR in neural and glial progenitor cells, either via genetic manipulations or by treatment with exogenous ligand, induces aberrant proliferation of cells with glial features, and can lead to the formation of tumor-like lesions [29–32].

The constitutive activation of EGFR conferred by EGFRvIII results in altered signaling relative to ligand-mediated activation of the full-length receptor with respect to activated downstream pathways, intracellular signaling molecules, and epigenetic regulators [33–35]. Complex cross-talk between signaling pathways in human GBM and GBM cell lines can complicate the investigation of activation of a single RTK. Using an immune competent murine model for glioma, based on the genetic manipulation of neural progenitor cells and driven by EGFRvIII, we identify the activity level of EGFRvIII as a determinant of tumor cell aggressiveness.

## RESULTS

### Generating tumor progenitor cells with differences in EGFRvIII activation

**Figure 1:**
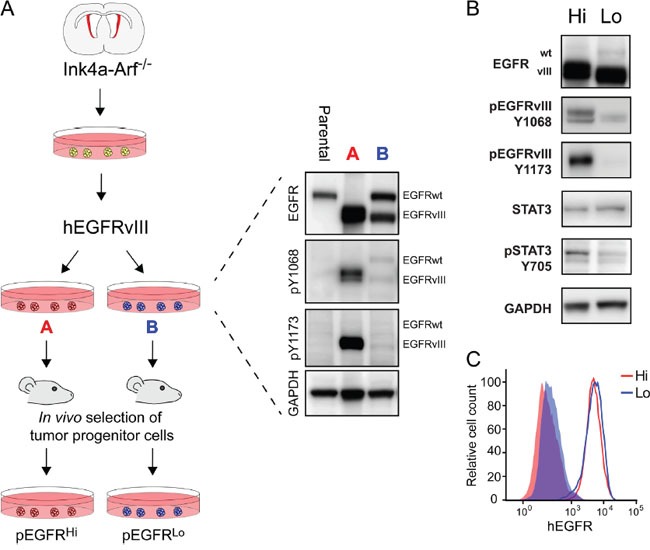
Generation of murine tumor cells with divergent EGFRvIII activity **A.** Paradigm for generating murine tumor progenitor cells with divergent EGFR activation from Ink4a/Arf-null neural progenitor cells and EGFRvIII transduction. After transduction, EGFRvIII-transduced cells were assayed for EGFR expression and activity level using western blot and were subsequently passaged through syngeneic mice as intracranial tumors to create the pEGFR^Hi^ and pEGFR^Lo^ lines. **B.** Western blot analysis of pEGFR^Hi^ (Hi) and pEGFR^Lo^ (Lo) demonstrating differences in EGFRvIII phosphorylation at pY1068 and pY1173 and Stat3 phosphorylation at pY705. **C.** Analysis of EGFRvIII surface levels on pEGFR^Hi^ (Hi) and pEGFR^Lo^ (Lo) cells using flow cytometry. Solid histograms represent negative controls. Data representative of triplicate experiments.

To generate tumor progenitor cell lines exhibiting divergent EGFRvIII activation, we used a model for high grade astrocytoma developed in mice [36]. In this model neural progenitor cells (NPCs) are isolated from the subventricular zone of Ink4a/Arf null mice and transduced with the human EGFRvIII. This and similar models making use of neural stem cells and EGFRvIII overexpression are known to produce tumors heterogeneous in growth and survival [37, 38]. Lysates from EGFRvIII-transduced cells derived from the same pool of parental NPCs revealed two lines with divergent EGFRvIII activity as determined by Western blotting, denoted line A and line B (Figure 1A). While both cell lines had increased EGFR activity relative to parental NPCs, only line A with high EGFRvIII activity had a decrease in expression of the full-length endogenous EGFRwt. To generate tumor progenitor cells, the two EGFRvIII-transduced lines were transplanted intracranially into syngeneic mice and the resulting tumors were propagated as tumorspheres in minimal essential media *in vitro* as pEGFR^Hi^ and pEGFR^Lo^, respectively (Figure 1A).

Following *in vivo* passage and selection of tumor progenitor cells, the relative differences in EGFR activity were preserved. pEGFR^Hi^ had increased abundance of phosphorylated EGFRvIII, as evidenced at Y1173 and Y1068 tyrosine residues compared to pEGFR^Lo^ (6.5- and 2.86-fold p-EGFR/total, respectively; p<0.005, n=4). Differences in EGFRvIII activation were not due to differences in total expression of EGFRvIII or differences in cell surface expression of EGFRvIII (Figure 1B and 1C). pEGFR^Hi^ also had increased STAT3 activation (6.89-fold; p>0.001, n=4), based on Y705 tyrosine residue phosphorylation (Figure 1B).

### EGFRvIII activity associated with more aggressive tumors and gene expression signature

**Figure 2:**
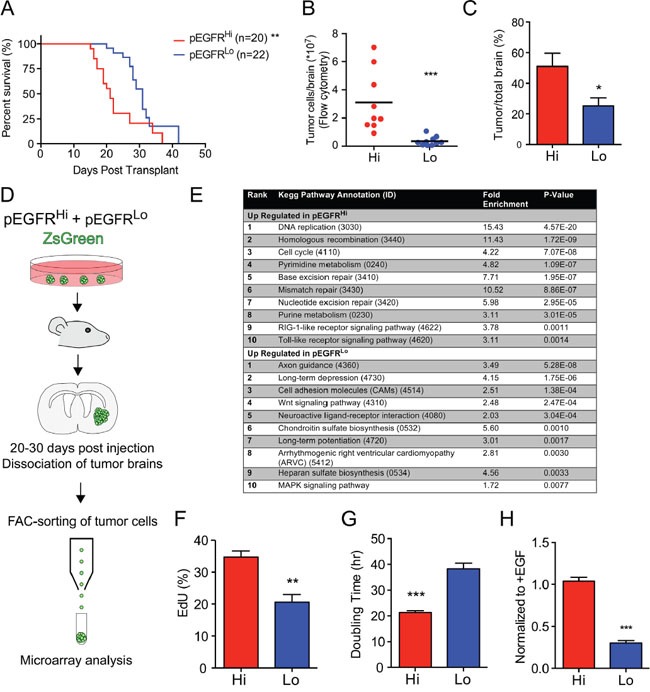
EGFRvIII activity associated with more aggressive tumors and gene expression signature **A.** Kaplan-Meier survival curve comparing survival of mice transplanted with pEGFR^Hi^ and pEGFR^Lo^ tumor cells. Log-rank test median survival 21 versus 31 days, respectively (p<0.0001) **B.** Total number of ZsGreen-tagged tumor cells per brain enumerated by fluorescence-activated cell sorting (FACS). **C.** Area of brain involved by tumor as a percentage of total brain area per section with the greatest tumor. **D.** Schematic of isolation of tumor cells by FACS for microarray analysis. **E.** Enrichment of KEGG pathway processes in pEGFR^Hi^ versus pEGFR^Lo^ tumors based on *in vivo* microarray analysis of gene expression in sorted cells. **F.** *In vitro* EdU incorporation in pEGFR^Hi^ and pEGFR^Lo^ following a 2-hour pulse. **G.** *In vitro* doubling time of pEGFR^Hi^ (Hi) and pEGFR^Lo^ (Lo) cells. **H.** Growth of pEGFR^Hi^ (Hi) and pEGFR^Lo^ (Lo) cells in the absence of added EGF ligand relative to growth in EGF-supplemented controls. p<0.05 = *, p<0.01 = **, p<0.001 = ***. Error bars are displayed as standard error of mean (SEM). (C) n=4.; (F and H) n=3; (G) pEGFR^Hi^ n=54, pEGFR^Lo^ n=37.

Orthotopic transplants of pEGFR^Hi^ and pEGFR^Lo^ revealed significant differences in tumor growth *in vivo*. While both groups developed signs of tumor by 42 days post transplant, the median survival of mice harboring pEGFR^Hi^ cells was 21 days versus 31 days for pEGFR^Lo^ cells (p<0.0001, n=6) (Figure 2A). The more aggressive pEGFR^Hi^ resulted in greater tumor burden than pEGFR^Lo^ even at comparable median survival time points. Enumeration of ZsGreen tagged tumor cells by fluorescence-activated cell sorting (FACS), revealed an 8.7-fold increase in pEGFR^Hi^ versus pEGFR^Lo^ tumor cells at respective median survival time points (p=0.001, n=6) (Figure 2B) and increased tumor area (p<0.05) (Figure 2C).

To investigate *in vivo* gene expression differences associated with increased EGFRvIII activity, fluorescently-tagged tumor cells were isolated by FACS from tumors and differential gene expression was determined by microarray analysis at median survival ±1 day (Figure 2D). KEGG pathway annotation of differentially expressed genes identified enrichment of processes related to proliferation and DNA repair in pEGFR^Hi^ tumors. Conversely, processes associated with cell-matrix interactions and the glycocalyx were enriched in pEGFR^Lo^ tumor cells, including ‘cell adhesion molecules’, ‘chondroitin sulfate biosynthesis’, and ‘heparan sulfate biosynthesis’. In addition, processes associated with a wider range of differentiation phenotypes, such as ‘axon guidance’ and ‘long-term potentiation’, were also enriched in pEGFR^Lo^ (Figure 2E).

Increased *in vivo* tumor burden, as defined by increased tumor cell number (Figure 2B), increased tumor area (Figure 2C), and enrichment of genes involved in DNA replication (Figure 2E) suggested increased proliferative capacity in pEGFR^Hi^ versus pEGFR^Lo^ cells. *In vitro*, pEGFR^Hi^ had increased incorporation of EdU (p<0.01) and greatly reduced doubling time compared to pEGFR^Lo^ (Figure 2F and 2G), reflecting an increased rate of proliferation. No differences in apoptosis were appreciated based on immunohistochemical analysis of cleaved caspase 3 in tumors (data not shown).

While EGFRvIII lacks a ligand-binding domain and can be activated independent of EGF ligand, EGFRvIII can act as a substrate for EGF-activated full-length EGFR [39]. To assess the role for EGF ligand-mediated signaling in the two lines, cell growth was compared in the absence of exogenous EGF ligand. While pEGFR^Hi^ maintained a high rate of proliferation, the growth of pEGFR^Lo^ was decreased three-fold in the absence of EGF (Figure 2H). These data suggest that the higher expression levels of full-length EGFR in pEGFR^Lo^ may confer some degree of ligand-mediated signaling in these cells that is not appreciated in pEGFR^Hi^.

### Blocked differentiation in EGFRvIII highly activated tumor progenitor cells

As compared to pEGFR^Hi^ cells, pEGFR^Lo^ cells had enriched expression of pathways associated with a more differentiated phenotype. Given that activation of EGFR can drive proliferation of a neural progenitor cells we examined the expression of a panel of genes associated with neural stem and progenitor cell maturation.

**Figure 3:**
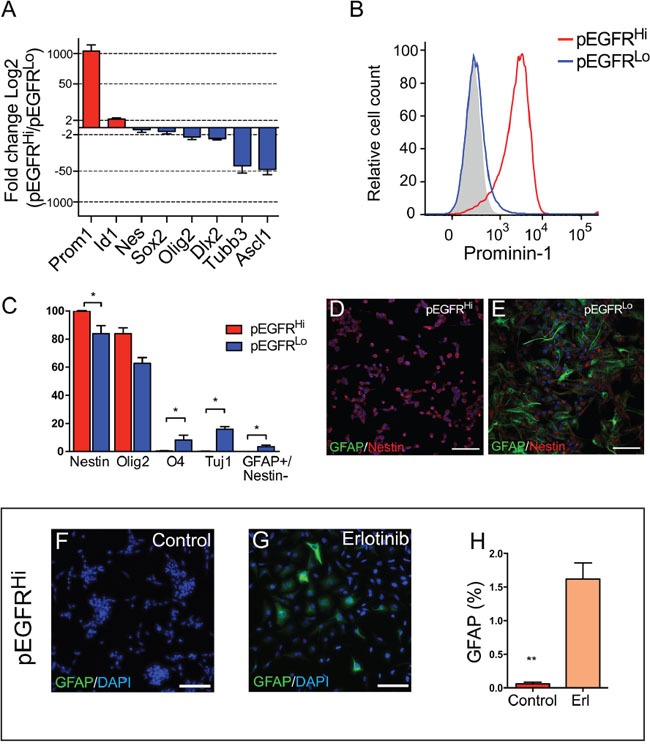
High EGFRvIII activity is associated with an immature stem cell phenotype and EGFRvIII-dependent block in differentiation **A.** Gene expression of neural stem and progenitor lineage markers in pEGFR^Hi^ relative to pEGFR^Lo^ cells by real-time quantitative PCR. **B.** Cell surface Prominin-1 expression in pEGFR^Hi^ (red) and pEGFR^Lo^ (blue) cells. Negative control in gray. Data representative of triplicate experiments. **C.** Protein expression of neuronal and glial differentiation markers 3 days after differentiation in pEGFR^Hi^ and pEGFR^Lo^ cells. **D** and **E.** Representative images of GFAP (green) and nestin (red) expression after 7 days of differentiation of pEGFR^Hi^ (D) and pEGFR^Lo^ cells (E). **F** and **G.** Representative images of GFAP (green) and nestin (red) expression after 7 days of differentiation of pEGFR^Hi^ control treated (F) or erlotinib treated (G) cells. **H.** Percentage of GFAP-expressing pEGFR^Hi^ cells after 7 days of differentiation in control or erlotinib-treated cells. p<0.05 = *. Scale bars D, E, F, G, = 50 μm. (A) n=3; (C) n=4; (H) n=4.

Real-time quantitative PCR demonstrated increased expression of the immature stem cell markers *Prom1* and *Id1* in pEGFR^Hi^ (Figure 3A). Conversely, expression of genes associated with cellular differentiation, such as *Tubb3* and *Ascl1* (Mash1), were upregulated more than 40-fold in pEGFR^Lo^. *Olig2* and *Dlx2* transcripts, genes commonly expressed by progenitor cells, were also more highly expressed in pEGFR^Lo^ compared to pEGFR^Hi^ (Figure 3A). Consistent with a more undifferentiated phenotype, more than 95% of EGFRvIII-activated pEGFR^Hi^ tumor cells expressed Prominin-1 on their cell surface (Figure 3B). In contrast, less than 2% of pEGFR^Lo^ tumor cells expressed Prominin-1 (Figure 3B).

In cell differentiation assays increased EGFRvIII activity in pEGFR^Hi^ cells was associated with reduced differentiation potential relative to in pEGFR^Lo^ cells. The pEGFR^Hi^ cells retained expression of nestin, a marker of immature neural stem and progenitor cells, and had decreased expression of glial (O4 and GFAP) and neuronal lineage markers (TUJ1/βIII-Tubulin) as compared to pEGFR^Lo^ cells (Figure 3C). Even after 7 days of culture under differentiation conditions pEGFR^Hi^ cells appeared largely undifferentiated, and displayed a small round cell-appearance with robust expression of nestin (Figure 3D). pEGFR^Lo^ cultures, in contrast, contained a significant population of GFAP-expressing cells, indicative of glial differentiation, and exhibited a bipolar or multipolar morphology (Figure 3E).

To determine whether the lower differentiation potential of pEGFR^Hi^ cells was due to high EGFR activation, we examined differentiation in the presence or absence of the EGFR tyrosine kinase inhibitor erlotinib. After 7 days of culture, pEGFR^Hi^ cells with EGFR inhibition exhibited increased GFAP expression and altered cell morphology, with more elongate cells, as compared to control-treated pEGFR^Hi^ cells, which retained their rounded, clumped morphology (Figure 3F-3H).

### Multicellular co-option of the vasculature in EGFRvIII activated tumors

**Figure 4:**
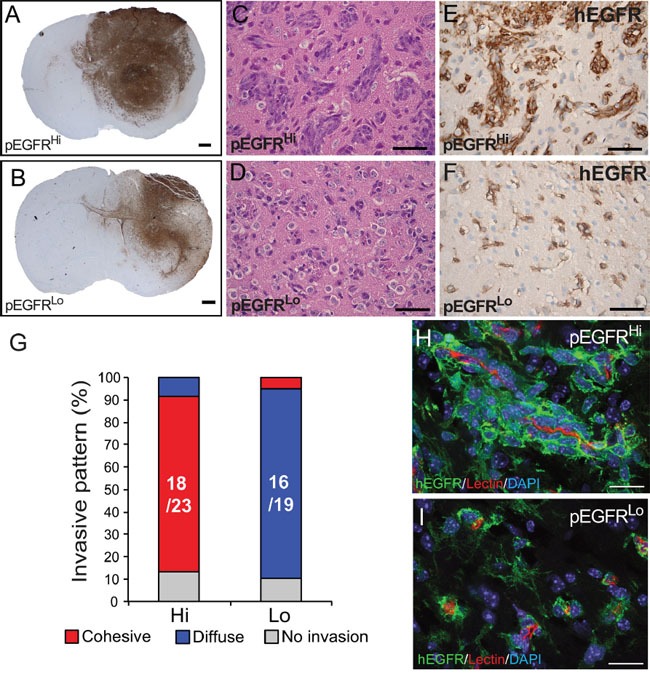
Blood vessel co-option and cohesive versus single cell invasion of tumor cells *in vivo* **A** and **B**. pEGFR^Hi^ (A) and pEGFR^Lo^ (B) form large, invasive tumors highlighted by hEGFR immunohistochemisty. At the infiltrating tumor edge, pEGFR^Hi^ **C** and **E.** tumor cells form cohesive clusters of invading tumor cells while pEGFR^Lo^ **D** and **F.** tumor cells exhibit diffuse, single cell invasion as demonstrated by representative images of H&E stained (C and D) and hEGFR immunolabeled tumor cells (E and F). **G.** Stability of cohesive and diffuse invasive phenotype in pEGFR^Hi^ (Hi) and pEGFR^Lo^ (Lo) tumors, respectively. Chi-square test for difference in invasive pattern p<0.0001. **H** and **I.** Confocal images of blood vessel co-option by pEGFR^Hi^ (H) and pEGFR^Hi^ (I) tumor cells *in vivo*. Tumor cells labeled by hEGFR (**green**), blood vessels by isolectin IB4 (**red**), and nuclei by DAPI (**blue**). Scale bars: A and B = 500 μm, C-F = 50 μm, H and I = 100 μm.

Both pEGFR^Hi^ and pEGFR^Lo^ tumors were highly infiltrative with involvement of the striatum, cerebral white matter, and cortex (Figure 4A and 4B), yet the pattern of tumor infiltration was different. At the invasive front, pEGFR^Hi^ tumors formed dense multicellular invasive cords of tumor cells (Figure 4C and 4E). In contrast, pEGFR^Lo^ tumors appeared diffusely infiltrative primarily as single cells (Figure 4D and 4F). The pattern of invasion was highly stable as 78% (18/23) of pEGFR^Hi^ and 84% (16/19) of pEGFR^Lo^ secondary intracranial tumors retained the pattern (Figure 4G). At the invasive tumor front, co-labeling of tumor cells and blood vessels revealed blood vessel co-option in both pEGFR^Hi^ and pEGFR^Lo^ tumors (Figure 4H and 4I). Multicellular clusters of pEGFR^Hi^ tumor cells were often identified in close association with the vasculature.

**Figure 5:**
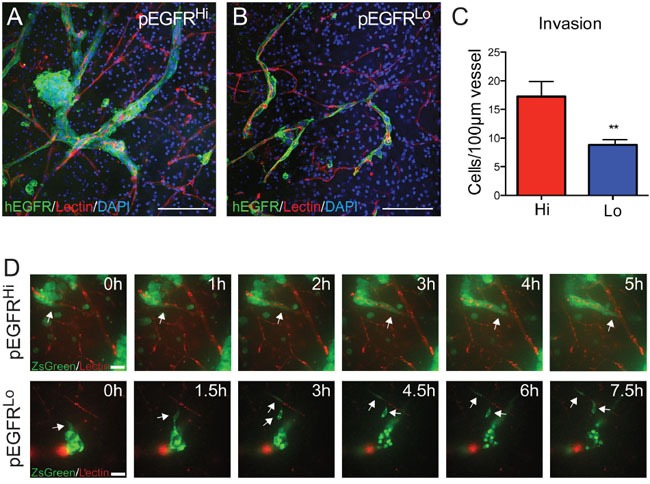
*Ex vivo* slice cultures demonstrate blood vessel co-option and preferential multicellular clustering by pEGFR^Hi^ **A** and **B.** Blood vessel co-option by both pEGFR^Hi^ and pEGFR^Lo^ tumor cells. At 24 hours after injection pEGFR^Hi^ cells (A) appear as cohesive clusters while pEGFR^Lo^ cells (B) exhibit a single-cell pattern. **C.** Blood vessel co-option in slice cultures quantified as tumor cell number/100 μm of blood vessel in pEGFR^Hi^ and pEGFR^Lo^ slice cultures at 24 hours. **D.** Time-lapse confocal imaging of invasion in brain slice cultures over 24 hours. Arrows highlight invading cells. Tumor cells labeled by hEGFR (A and B) and ZsGreen (D) (**green**), blood vessels by isolectin IB4 (**red**), and nuclei by DAPI (**blue**). Scale bars: A and B = 100 μm, D = 20 μm. p<0.01 = **. (C) pEGFR^Hi^ n=6, pEGFR^Lo^ =7. Error bars are displayed as standard error of mean (SEM).

To model these invasive behaviors *ex vivo* we injected tagged tumor cells into freshly isolated brain slices and performed confocal imaging of fluorescently-tagged tumor cells and lectin-labeled blood vessels. At 24 hours both pEGFR^Hi^ and pEGFR^Lo^ cells readily invaded into brain slices and showed preferential co-option of blood vessels (Figure 5A and 5B). Similar to their respective *in vivo* patterns of invasion, pEGFR^Hi^ cells generated multicellular clusters (Figure 5A) while pEGFR^Lo^ cells tended to extend as single cells along the vasculature (Figure 5B). Quantification demonstrated nearly twice as many blood vessel-associated tumor cells per 100 μm length of vessel in pEGFR^Hi^ compared to pEGFR^Lo^ (Figure 5C). To distinguish multicellular invasion from local tumor cell proliferation in the highly proliferative pEGFR^Hi^ cells, we performed time-lapse confocal imaging of tumor cells in brain slice cultures over 24 hours. Both pEGFR^Hi^ and pEGFR^Lo^ tumor cells invaded along blood vessels, but only pEGFR^Hi^ tumor cells invaded as multicellular groups of cells (Figure 5D, Suppl video 1 (pEGFR^Hi^) and 2 (pEGFR^Lo^)).

### Decreased cell adhesion and invasion in EGFRvIII highly activated tumor cells

**Figure 6:**
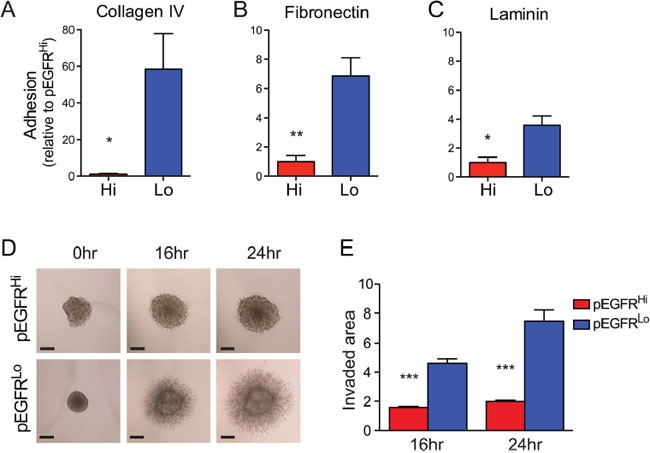
EGFRvIII highly active tumor cells exhibit decreased cell adhesion and invasion *in vitro* **A**-**C.** Adhesion of pEGFR^Hi^ and pEGFR^Lo^ cells to collagen IV (A), fibronectin (B), and laminin (C), matrices, expressed as fold-change relative to pEGFR^Hi^. **D.** Representative images of matrigel 3D spheroid invasion assay and quantification of pEGFR^Hi^ and pEGFR^Lo^ invasion **E.** at 16hr and 24hr normalized to pEGFR^Hi^ at 0 hours. Scale bars: A=50 μm. p<0.05 = *, p<0.01 = **, p<0.001 = ***. Error bars are displayed as standard error of mean (SEM). (A) n=4, (B and C) n=3, (E) n=4.

Differences in *in vivo* and *ex vivo* invasion exhibited by pEGFR^Hi^ and pEGFR^Lo^ tumor cells suggested EGFRvIII activity may influence cell-ECM interactions. Tumor cells plated on cell culture plates pre-coated with laminin, collagen IV, or fibronectin revealed a marked difference in cell adhesion between pEGFR^Hi^ and pEGFR^Lo^. Increased EGFRvIII activity was associated with a reduced ability to adhere to all substrates (Figure 6A-6C). To assess how decreased cell adhesion may influence invasion in a 3D matrix we utilized an *in vitro* matrigel invasion assay in which the primary components are laminin, collagen IV, and enactin/nidogen-1 [40, 41]. While pEGFR^Lo^ tumor cells were highly invasive even by 16 hours, pEGFR^Hi^ showed very limited invasion even after 24 hours (Figure 6D-6E). Thus, the increased EGFRvIII activity in pEGFR^Hi^ was associated with reduced adhesion and reduced invasion *in vitro*.

**Figure 7:**
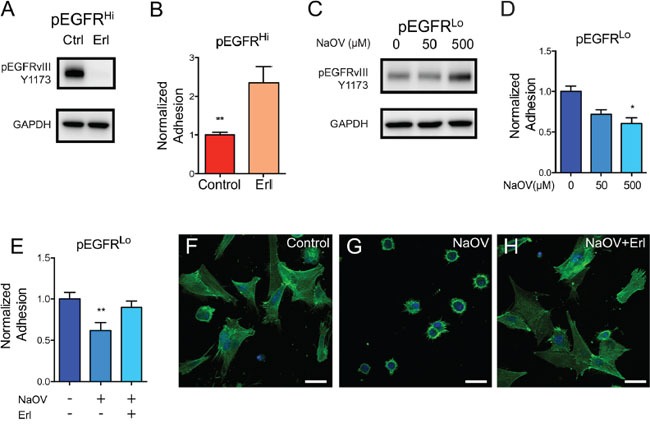
EGFRvIII activity dictates adhesion properties in mouse tumor cells **A.** Western blot analysis of EGFR activation, as demonstrated by phosphorylation at Y1173, in control (Ctrl) and erlotinib (Erl) treated pEGFR^Hi^ tumor cells. **B.** EGFR inhibition increases adhesion of pEGFR^Hi^ cells to a collagen IV-coated substrate. **C.** Phosphorylation of EGFR at Y1173 in pEGFR^Lo^ cells treated with 0, 50 μM, and 500 μM of the phosphatase inhibitor sodium orthovanadate (NaOV). **D.** Reduced adhesion of pEGFR^Lo^ cells to collagen IV in the presence of increasing concentrations of NaOV. **E.** NaOV-induced reduction in adhesion to collagen IV is restored by inhibition of EGFR by Erl in pEGFR^Lo^ cells. **H**-**J.** Representative confocal images of phalloidin labeled f-actin (green) and nuclei (blue) in control (H), NaOV (I), and NaOV+erlotinib (J) treated pEGFR^Lo^ cells 2 hours after plating. p<0.05 = *, p<0.01 = **. Error bars are displayed as standard error of mean (SEM). (B) n=6, (D) n=3, (E) n=8. Scale bars F-H = 20 μm. (B, D, E) Representative data from triplicate experiments.

To investigate whether reduced adhesion was due to increased EGFRvIII activity, cell adhesion assays were performed while modulating EGFR activity. Reduction of EGFR activity in pEGFR^Hi^ cells, using a 2-hour treatment with erlotinib, demonstrated a 2.35-fold increase in adhesion to collagen IV (p<0.01, n=6) (Figure 7A-7B). In contrast, adhesion was decreased when EGFR activity was acutely increased in pEGFR^Lo^ cells using the phosphatase inhibitor sodium orthovanadate (NaOV) (Figure 7C-7D). Importantly, pEGFR^Lo^ cell adhesion could be restored upon inhibition of EGFR in NaOV-treated cells (Figure 7E). This increase in pEGFR^Lo^ adhesion was accompanied by the restoration of well-organized stress fibers in treated cells (Figure 7F-7H).

## DISCUSSION

GBM are characterized by their heterogeneity, including tumor genetics [6], invasive phenotype [7], and RTK signaling activation [8]. This heterogeneity is an important prognostic factor in GBM and presents a great therapeutic challenge [42]. Murine models for GBM provide a controlled genetic background to study individual oncogenic events [43], however, there has not been a major focus on modeling and studying the impact of differences in RTK activity levels [37, 38, 44, 45]. The potential importance of RTK activity on disease is highlighted by elegant studies examining the cross-talk between constitutive versus ligand-dependent EGFR signaling. Ligand-induced activation of endogenous EGFR phosphorylates EGFRvIII and promotes STAT3 activation, but can also disrupt EGFRvIII-induced NFkB signaling [39, 46]. Moreover, ligand-independent activation of EGFR results in non-canonical EGFR signaling through the formation of an EGFR, TBK1, and IRF3 complex, downstream effects of which include reduced sensitivity to chemotherapy [47]. Using a murine model for glioma, we demonstrate EGFRvIII activity-dependent regulation of cell differentiation and cell adhesion and associate these differences with altered animal survival and *in vivo* tumor growth and invasion. Our results suggest that the activity of the EGFR signaling pathway, in addition to the specific genetic alteration, is an important determinant of tumor aggressiveness.

The more aggressive pEGFR^Hi^ tumors exhibited both increased proliferation and a more cohesive pattern of invasion as compared to pEGFR^Lo^ tumors. While increased proliferation is a known driver of malignancy, differences in invasive pattern may also contribute. Indeed, tumor progression following antiangiogenic therapy has been associated with a perivascular pro-invasive phenotype [48, 49].

Diffuse invasion of tumors cells is a characteristic feature of human GBM and contributes to poor prognosis, yet the pattern of invasion is heterogeneous [7] suggesting the contribution of multiple factors. Even within a single patient several patterns can often be observed and differences in cell adhesion, proteolysis, and migration may all contribute. Blood vessel co-option was prominent in pEGFR^Hi^ and pEGFR^Lo^ tumors, yet pEGFR^Hi^ invaded as multicellular clusters while the less aggressive pEGFR^Lo^ invaded as single cells. Single cell invasion of pEGFR^Lo^ was associated with increased *in vitro* adhesion and increased *in vivo* expression of genes implicated in cell-matrix interactions, including integrins and proteoglycans. In pEGFR^Hi^, inhibition of EGFR increased cell-matrix adhesion and promoted a more differentiated phenotype. While the identification of factors downstream of EGFR that mediate cohesive invasion is a focus of future studies, it is notable that by gene expression array fibronectin (*FN1*) expression was elevated 5.8-fold in pEGFR^Hi^ tumors. FN1 has previously been implicated in cohesive invasion in glioma and gene knockdown increased cell-matrix adhesion [50]. In addition, differences in protease activity or activation of other receptor tyrosine kinases such as MET can have profound influences on tumor cell invasion [49, 51].

Abnormal activation of EGFR in neural stem and progenitor cells promotes proliferation and is detrimental to neuronal cell maturation [29–32]. pEGFR^Hi^ exhibited profound alterations in progenitor cell phenotype with high expression of Prominin-1 and decreased ability to differentiate, which was at least partially dependent on EGFR activation. In immortalized neural stem cells, EGFRvIII expression has previously been associated with decreased differentiation, increased focal adhesion disassembly, and perivascular invasion *in vivo* [52]. In addition, a CD133-expressing subset of EGFRvIII^+^ glioma cells was shown to have increased self-renewal and tumorigenicity [53]. While several factors, including homozygous deletion of Ink4a/Arf^−/−^ [37], likely contribute to the overall modest degree of cell differentiation that we observed, our data strengthen the link between abnormal EGFR signaling pathway activity and aberrations in cell differentiation.

EGFRvIII is known to be expressed at varying levels within a tumor [54, 55] and the regulation of extrachromosomal EGFRvIII DNA is a major mechanism of treatment resistance [56]. Our data, in a genetically controlled background, suggest that the level of EGFR activation, not just the total protein levels, is an important determinant of tumor cell phenotype. Given the complexity of EGFR signaling and the multiple feedback loops and regulatory mechanisms [57], several factors may contribute to differences in EGFR activity and ultimately cellular behavior. These include differences in phosphatase activity, cell differentiation [58], receptor endocytosis, and the activity of other receptor tyrosine kinases [57]. Our data emphasize the importance of signaling pathway activity, in addition to the genetic status of EGFR, in tumor cell behavior and aggressiveness.

## MATERIALS AND METHODS

### Cell culture conditions and reagents

Murine neural stem cell and tumorspheres were cultured as described previously [36, 59]. Three dimensional invasion assays were performed as previously described [60]. EGF-dependent growth was calculated over three consecutive passages comparing cells cultured in defined neurosphere media including fibroblast growth factor in the presence and absence of added EGF (20 ng/ml). For adhesion assays, wells of a 96-well plate were coated with 2 μg/cm^2^ Laminin (Sigma L2020), 10 μg/cm^2^ Collagen IV (Sigma C5533) or 5 μg/cm^2^ Fibronectin (Sigma F1141). 1-5×10^4^ cells were plated per well and incubated for 2 hours. For assays involving erlotinib and sodium orthovanadate (NaOV) drug treatments, 1 μM erlotinib, 50 μM NaOV, or 500 μM NaOV was added to cell suspension for the 2 hour duration of the assay. For adhesion assays using co-treatment of erlotinib and NaOV, 1 μM erlotinib and 250 μM NaOV was used. Protein lysates were prepared as previously described [36]. Protein lysates for studying effects of erlotinib and NaOV on EGFR phosphorylation were acquired after 2 hours of incubation in sphere suspension cultures. Images acquired for invasion and adhesion assays were acquired using a DMI IL LED Leica microscope (Leica, Germany) and quantified using ImageJ 1.48v (http://imagej.nih.gov/ij/). All antibodies used are described in Table 1.

**Table 1:** Antibodies used

Antibody	Manufacturer	Clone	Catalog no	Application	Concentration
Ms α Phospho EGFR Y1068	R&D Systems	338324	MAB3570	WB	1:500
Rb α Phospho EGFR Y1173	Cell Signaling	53A5	4407	WB	1:500
Rb α Phospho Stat3 Y705	Cell Signaling	D3A7	9145	WB	1:1000
Ms α Stat3	Cell Signaling	124H6	9139	WB	1:1000
GAPDH	Millipore	6C5	MAB374	WB	1:10000
Dk α Rb HRP	GE Healthcare		NA9310V	WB	1:5000
Dk α Ms HRP	GE Healthcare		NA934V	WB	1:5000
Ms α hEGFR	Dako	H11	M3563	IF, IHC	1:200
Isolectin IB_4_-568	ThermoFisher Scientific		I21412	IF and live	1:200
Rb α EGFR	Santa Cruz Biotechnology		Sc-03	WB	1:200
Ms α Nestin	EMD Millipore	rat401	MAB353	IF	1:200
Rb α GFAP	Dako		Z0334	IF	1:400
Ms α Tuj1	Covance		PRB435P	IF	1:400
Rb α Olig2	EMD Millipore		AB9610	IF	1:500
Ms α O4	EMD Millipore		MAB345	IF	1:100
Gt α Rb Alexa 488	ThermoFisher Scientific		A-11008	IF	1:500
Gt α Ms Alexa 568	ThermoFisher Scientific		A-11004	IF	1:500

Abbreviations: Ms, mouse; Rb, rabbit; Dk, donkey; Gt, goat; WB, Western blotting; IF, immunofluorescence; IHC, immunohistochemistry

### Tumor cell isolation and RNA isolation

Tumor bearing brains were harvested from six mice with each tumor type and the cerebellum and olfactory bulbs were removed. The brains were diced finely with a razor, dissociated using Worthington's Papain Dissociation System (LK003150) and passed through a 70um filter to obtain a single cell suspension. To remove myelin and red blood cells the cell suspension was re-suspended in 35% Percoll^TM^ (GE Healthcare) and underlaid with 70% Percoll^TM^. Following a 30 minute centrifugation purified cells were removed from the interface between the 35% and 70% layers and counted for viability. ZsGreen+ tumor cells were isolated by FACS using a BD FACSAria III into TRIzol Reagent (Ambion Life Technologies, Japan), using Dapi to exclude dead cells. Sorted cells were re-suspended in TRIzol Reagent (Ambion Life Technologies, Japan) and RNA was purified using the RNeasy Mini Kit (Qiagen, Germany) according to the manufacturers protocol.

### *In vivo* transplantation

Dissociated cells were transplanted as previously described previously [36, 59]. FVB mice (Charles River Laboratories, Wilmington, MA) were used for tumor microarray profiling experiments.

### Immunohistochemistry

Murine tumor tissue for immunohistochemical analysis was collected at first sign of tumor growth by perfusing mice with 4% paraformaldehyde and postfixed overnight in 4% PFA. Following postfixation tissue was rinsed in PBS and stored in 70% ethanol until further processing. Murine tumor paraffin embedded sections were immunostained for hEGFR (Dako) to label EGFRvIII-expressing tumor cells. Immunohistochemistry was performed according to standard protocols on the Ventana Medical Systems Benchmark XT (Ventana Medical Systems Inc. Tucson, AR, USA). Histological analysis was performed on hematoxylin and eosin stained sections. Tumor area was analyzed on hEGFR-labeled sections and quantified in ImageJ 1.48v (http://imagej.nih.gov/ij/).

### Ex vivo invasion and time-lapse imaging

Brains from P7-P21 FVB mice were dissected, put in cold aCSF, sectioned using a HM650V vibratome (Thermo Fisher scientific, San Jose, CA) at thickness of 190 μm. Slices were incubated in RT aCSF for 30 mins prior to transfer to wells with a MilliCell-CM 40 μm membranes (Millipore, Bedford, MA). For live imaging, ZsGreen expressing cells were injected underneath the slice using a Hamilton syringe (Hamilton, Reno, NV), for confocal analysis cells were deposited on top of slices using P10 pipette. For live imaging slices were imaged after 2 hours of incubation at 37°C, using a Zeiss Observer Z1 equipped with a Zeiss Observer XL stage incubator (37°C, 5% CO_2_, humidified air) (Carl Zeiss, Germany) and a Yokogawa spinning disc scanning unit (Yokogawa electric, Japan). Movies assembled using Zeiss imaging software (Carl Zeiss) at a single plane from images acquired over 6-8 hours. For confocal analysis, slices were fixed using 4% paraformaldehyde in phosphate-buffer saline 24 hours after tumor cell deposition, for 4-6 hours. A proteinase K antigen retrieval step was used to image hEGFR labeled tumor cells, followed by 1 hour of block/permabilization, 24-72 hours incubation in primary anti-human EGFR antibody antibody (Dako, Denmark, Clone H11) at 4°C, and 2 hours incubation in secondary antibodies. Slices were washed in PBS 3-5 times after each antibody incubation step. Slices were mounted onto Superfrost plus glass slides and mounted using Dako fluorescent mounting media (Dako, Denmark). Images were acquired on a Zeiss LS780 laser confocal microscope (Carl Zeiss).

### Differentiation assay

Cells were prepared as a single cell suspension in normal culture media, 500-1000 cells per well were added to a laminin/poly-d-lysine coated 8-well culture slide (BD Biocoat 354688, BD biosciences) in 300 μL growth media. The culture slides were incubated overnight and the media carefully removed and replaced with 300 μL of neurobasal (Thermo Fisher) + 2% FBS to induce differentiation. For studies using erlotinib, erlotinib (1 μM) or DMSO was included in differentiation media. Culture slides were incubated for 3 to 7 days, then fixed in 4% PFA. After immunolabeling, six images per well were taken on a Zeiss Axioimager M1 or a Zeiss LSM780 confocal microscope (Carl Zeiss), counted for positivity in ImageJ 1.48v and averaged.

### Visualization of f-actin cytoskeleton

pEGFR^Lo^ cells were fixed in 4% PFA 2 hours after seeding cells onto laminin/poly-d-lysine coated 8-well culture slides (BD biosciences). F-actin was visualized using Alexa fluor-546 phalloidin according to the manufacturer's instructions (Thermo Fisher). Cells were treated with DMSO, 250 μM NaOV, or 250 μM NaOV + 1 μM erlotinib for the 2 hour duration of the assay.

### Flow cytometry

Cell surface PE-conjugated mouse Prom1 antibody (Miltenyi Biotec, France, Clone MB9-3G8) and anti-human EGFR antibody (Dako, Denmark, Clone H11) were used to analyze Prominin-1 and EGFRvIII surface expression, respectively. Incubation using EGFR was followed by fluorescently tagged secondary incubation. Data was acquired using a FACSCalibur or FACSCanto flow cytometer (BD biosciences). For EdU incorporation assay spheres were cultured for 2 days, incubated with 2 μM EdU for 2 hours, and labeled using Click-IT EdU Assay kit (Thermo Fisher). Cells were analyzed using a FACSCalibur flow cytometer (BD biosciences). Data was analyzed using FlowJo 10 (Tree star, San Carlos, CA).

### RNA isolation, cDNA synthesis and RT-PCR

Cells isolated from mouse brains or dissociated tumorspheres from culture were prepared as a single cell suspension and re-suspended in an appropriate volume of RLT buffer from RNeasy mini kit (Qiagen, Germany). RNA was isolated as per the manufacturers instructions and quantified using a Nanodrop (Nanodrop Technologies Inc., Wilmington, DE) Spectrophotometer. Complementary DNA was synthesized using Oligo(dT) primers and Superscript III (18080-051, Thermo Fisher Scientific). Primers were synthesized by IDT (IDT Inc, Coralville, IA). The following primers were used: Id1 (Forward (Fw): CCTAGCTGTTCGCTGAAGGC, Reverse (Rv): GTAGAGCAGGACGTTCACCT), Prom1 (Fw: GACCAGGACTCGGATCAAAGG, Rv: TGTACTGCTCCACTACATAGTCA), Nes (Fw: AGAGTCAGATCGCTCAGATCC, Rv: GCAGAGT CCTGTATGTAGCCAC), Sox2 (Fw: GCGGAGT GGAAACTTTTGTCC, Rv: GGGAAGCGTGTACTT ATCCTTCT), Olig2 (Fw: TCCCCAGAACCCGA TGATCTT, Rv: CGTGGACGAGGACACAGTC), Dlx2 (Fw: CTACGGCACCAGTTCGTCTC, Rv: CCGTTCACT ATTCGGATTTCAGG), Tubb3 (Fw: TAGACCCC AGCGGCAACTAT, Rv: GTTCCAGGTTCCAAGTC CACC), Ascl1 (Fw: GCAACCGGGTCAAGTTGGT, Rv: CAAGTCGTTGGAGTAGTTGGG). Real Time PCR was performed on a 7900 HT Fast Real-Time PCR System (Thermo Fisher Scientific) using FastStart Universal SYBR Green Master (Rox) (049138500011, Roche, Switzerland). Ct values were normalized to GAPDH Ct values for each individual sample and then averaged across experiments.

### Microarray profiling and analysis

Expression profiling was conducted by the Gladstone Institute Genomics Core Facility using Affymetrix Mouse Gene 1.0 ST Array (901169). Raw expression data was RMA normalized and Log2 transformed using Affymetrix Expression Console Software. The microarray data have been deposited in the GEO database (accession number GSE87332) and described in accordance with MAIME guidelines. Multiple Experiment Viewer Software (MeV; http://mev.tm4.org/#/welcome) was used to perform Significance Analysis of Microarray (SAM) to determine differentially expressed genes between the two tumor types. A 1% false discovery rate and 2-fold expression difference was applied. The subsequent list of differentially expressed genes were analyzed for enriched Kegg Pathways using the Database for Annotation, Visualization and Integrated Discovery (DAVID).

### Ethical statement

All animal work was approved by the UCSF institutional care and use committee (IACUC), approval number AN105263.

### Statistics

All statistics were performed using GraphPad Prism 6.0 (GraphPad Software, San Diego, CA). The Mann-Whitney Rank Sum or the Student's t-test were used to determine differences between experimental groups as appropriate. The log-rank test was used to compare groups in the Kaplan-Meier survival analysis. Chi-squared test was used to compare the frequency of invasive phenotypes in Figure 1C.

## SUPPLEMENTARY VIDEOS


