# Supplementary material for: GBM heterogeneity as a function of variable epidermal growth factor receptor variant III activity
Source: Oncotarget. 2016 Oct 12;7(48):79101–16. doi: 10.18632/oncotarget.12600 (PMC5346701; doi:10.18632/oncotarget.12600)
Supplement: Supplementary file 1 [file oncotarget-07-79101-s001.pdf]

# GBM heterogeneity as a function of variable epidermal growth factor receptor variant III activity

## SUPPLEMENTARY VIDEOS

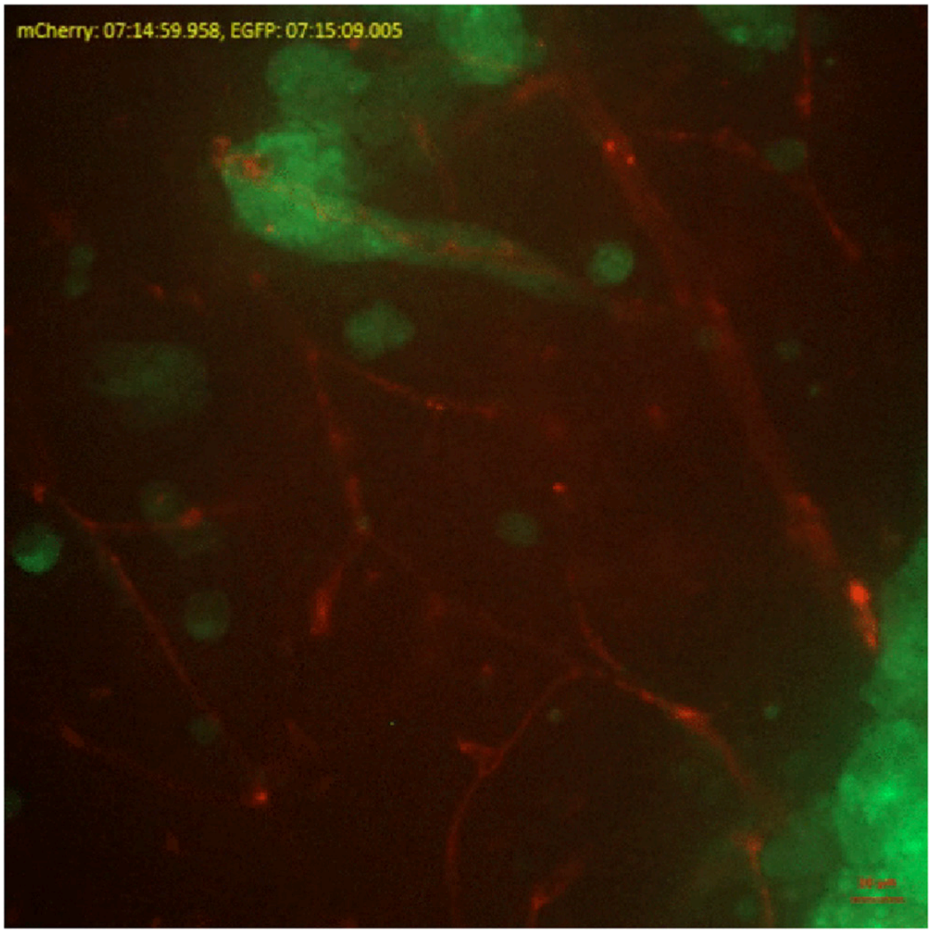


See Supplementary Video 1

**Supplementary Video S1: Ex vivo slice invasion of pEGFR-Hi cells.** ZsGreen-expressing pEGFR-Hi cells invading along lectin IB4-labeled vasculature in multicellular groups. Duration of acquisition: 5 hours. Scale bar = 20µm.

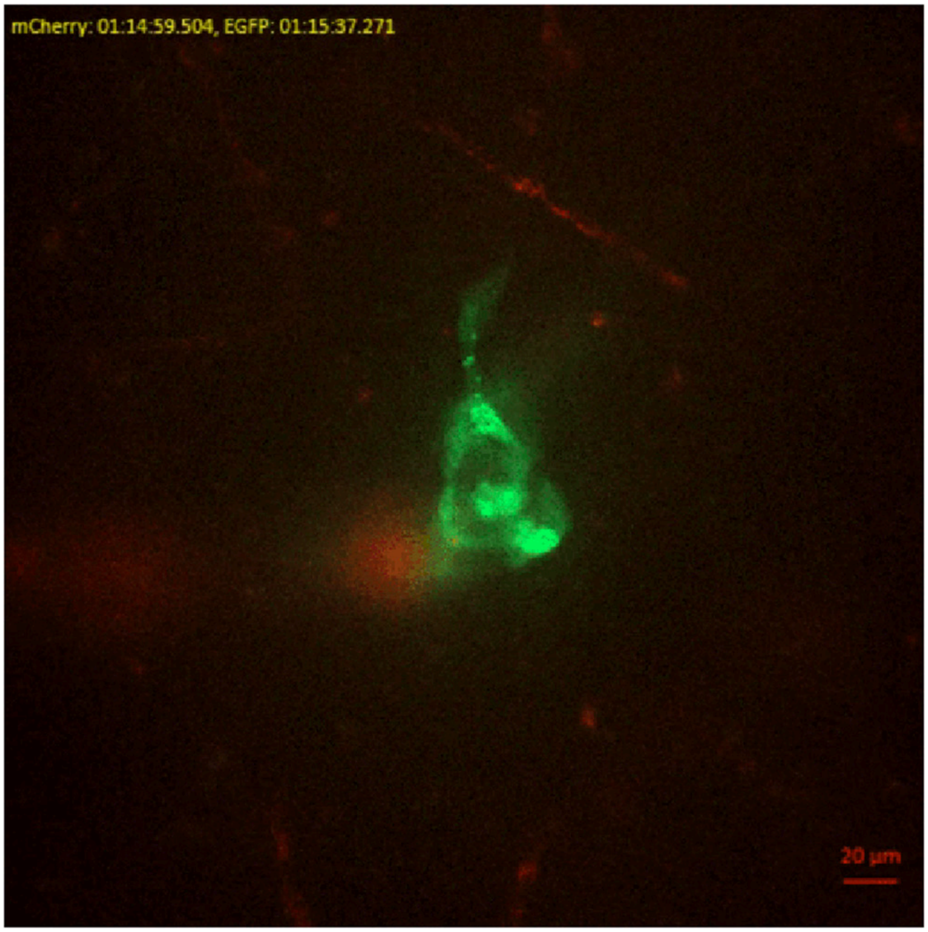


See Supplementary Video 2

**Supplementary Video S2: Ex vivo slice invasion of pEGFR-Lo cells.** ZsGreen-expressing pEGFR-Lo cells invading along lectin IB4-labeled vasculature as single cells. Duration of acquisition: 6.5 hours. Scale bar = 20µm.
